# Orthogonal proteomics methods to unravel the HOTAIR interactome

**DOI:** 10.1038/s41598-022-05405-6

**Published:** 2022-01-27

**Authors:** Louis Delhaye, Edith De Bruycker, Pieter-Jan Volders, Daria Fijalkowska, Delphine De Sutter, Sven Degroeve, Lennart Martens, Pieter Mestdagh, Sven Eyckerman

**Affiliations:** 1 Department of Biomolecular Medicine, Ghent University, Ghent, Belgium; 2 Center for Medical Biotechnology, VIB-UGent, Ghent, Belgium; 3 OncoRNALab, Center for Medical Genetics Ghent (CMGG), Ghent University, Ghent, Belgium; 4 Cancer Research Institute Ghent (CRIG), Ghent University, Ghent, Belgium

**Keywords:** Protein-protein interaction networks, Long non-coding RNAs

## Abstract

Accumulating evidence highlights the role of long non-coding RNAs (lncRNAs) in cellular homeostasis, and their dysregulation in disease settings. Most lncRNAs function by interacting with proteins or protein complexes. While several orthogonal methods have been developed to identify these proteins, each method has its inherent strengths and limitations. Here, we combine two RNA-centric methods ChIRP-MS and RNA-BioID to obtain a comprehensive list of proteins that interact with the well-known lncRNA HOTAIR. Overexpression of HOTAIR has been associated with a metastasis-promoting phenotype in various cancers. Although HOTAIR is known to bind with PRC2 and LSD1 protein complexes, only very limited unbiased comprehensive approaches to map its interactome have been performed. Both ChIRP-MS and RNA-BioID data sets show an association of HOTAIR with mitoribosomes, suggesting that HOTAIR has functions independent of its (post-)transcriptional mode-of-action.

## Introduction

Advances in high-throughput RNA-sequencing have shown that the human transcriptome is more complex than previously anticipated. Specifically, the number of non-coding genes has skyrocketed, which is unsurprising as protein-coding genes encompass only 2% of the human genome^1–3^. LncRNAs are transcripts that are over 200 nucleotides in length and do not code for a peptide or protein. LncRNAs are the bulk of the non-coding transcriptome in terms of number of genes. Although some lncRNAs, e.g. Xist and MALAT1, have been studied extensively and are functionally well characterized, most lncRNAs remain to be characterized both functionally and mechanistically. Dysregulated expression of lncRNAs has been shown for a wide spectrum of human diseases, making it important to better understand the role lncRNAs play in the molecular pathology of these diseases^4–6^. In addition, the highly tissue-specific expression profile of lncRNAs creates a novel source of potential targets for diagnostic, prognostic, and therapeutic applications to identify and treat human diseases^7^.

The molecular mechanisms underlying lncRNA function typically involve interactions with other biomolecular entities in the cell^8^. Some lncRNAs act as miRNA sponges, while others regulate gene expression by modulating protein complexes on regulatory regions in the genome or on mRNAs. Their generally low abundance makes it challenging to work with lncRNAs even in tissue culture conditions with human cell lines. Nevertheless, insights in these mechanisms are crucial for the translation towards clinical applications. In general, RNA-centric RNA–protein interactomics methods are based on affinity purification using biotinylated capture probes and various ways of RNA–protein crosslinking, e.g. iDRIP-MS^9^, ChIRP-MS^10^, or RAP-MS^11^, to enrich the target RNA and its associated interacting proteins, which are identified by subsequent mass spectrometry. Although conceptually very similar, methodological differences in iDRIP-MS, ChIRP-MS, and RAP-MS technologies have shown remarkable differences in size and content of the identified interactome of the lncRNA Xist, a crucial player in X-chromosome inactivation. All these studies have revealed Xist interacting proteins using different methods, all highlighting key interactors such as SPEN/SHARP and hnRNPU/SAF-A that are required for X-chromosome inactivation. Following the success of proximity labeling (PL) enzymes, such as BioID^12^ and APEX2^13^, in protein–protein interactions (PPI) studies, recently RNA interactomics studies have also implemented PL assays, e.g. RaPID^14^, RNA-BioID^15^, or CARPID^16^, to label RNA-associated proteins in their cellular context. In these methods, the PL enzyme is recruited to a specific RNA transcript using either an aptamer-based approach or a catalytically inactive RNA-targeting CRISPR system. We argue that, similar as in PPI studies, RNA interactome studies would benefit from the combination of orthogonal proteomics methods to generate a high confidence set of interacting proteins^17,18^. Braun et al.^19^ showed that combining orthogonal high-throughput binary assays can be used to score the confidence of PPIs. Therefore, it has become useful practice in the PPI field to combine AP-MS and PL assays to provide an in-depth mapping of a protein’s interaction network^17,18,20,21^.

HOTAIR (HOX Transcript Antisense Intergenic RNA) is a lncRNA expressed from the *HOXC* gene cluster during limb patterning in early development^22,23^. In addition, several studies have implicated an oncogenic role for HOTAIR in the proliferation and metastasis of various cancers. Rinn et al.^22^ and Tsai et al.^23^ showed that HOTAIR interacts with the polycomb repressive complex PRC2 as well as LSD1, a core member of the CoREST/REST demethylase complex. More recently, however, Portoso et al.^24^ have shown that PRC2 is dispensable for HOTAIR-mediated gene silencing, putting forth the question whether HOTAIR specifically recruits PRC2 or whether PRC2 is recruited to target genes as a consequence of HOTAIR-mediated gene silencing.

Although HOTAIR has been shown to have an oncogenic role in breast cancer cells, to our knowledge, an unbiased screen of the HOTAIR interactome has not yet been performed in a breast cancer cell line. Here, we set out to map the protein interactome of HOTAIR by combining data from both PL and ChIRP-MS experiments. Overlapping the results of both methods shows that HOTAIR potentially associates with MRPL proteins, although we demonstrate that this interaction does not occur within mitochondria.

## Material and methods

### Cell culture

HEK293T and Flp-In™ T-REx™ 293 (ThermoFisher Scientific R78007) cells were cultured in Dulbecco’s Modified Eagle Medium (DMEM) supplemented with 10% FBS (ThermoFisher Scientific #10270106). MCF7 (ATCC HTB-22) cells were cultured in Minimum Essential Medium Eagle (MEM) supplemented with 10% FBS, 1% non-essential amino acids, 1% HEPES, 1% sodium pyruvate, and 1% GlutaMAX™ (ThermoFisher Scientific #35050038). Parental cell lines were maintained in antibiotic-free conditions, experiments were performed in media supplemented with 30 U/mL Penicillin–Streptomycin. Cells were kept under 60–70% confluency and passaged twice a week. Cell lines were confirmed mycoplasma-free by using a mycoplasma PCR detection kit.

To generate a genomically stable MCP-BirA* cell line, 6 × 10^5^ FlpIn T-REx 293 cells were transfected with 2.3 µg pOG44 (ThermoFisher Scientific V600520) and 0.25 µg pDEST-MCP-BirA*-FLAG using Lipofectamine LTX according to the manufacturer’s instructions. Twenty-four hours post-transfection, cells were split to a confluency of 25% and the medium was supplemented with 15 µg/mL blasticidin and 50 µg/mL hyromycin. Transfected cells were maintained and passaged as needed under selection for two weeks until separate foci could be observed. Doxycycline-regulated expression of the MCP-BirA*-FLAG fusion protein was validated by western blot.

### Molecular cloning

The ORF of a tandem dimer MCP was amplified from pMS2-HB (Addgene #35573) with attB1 and attB2 Gateway sites included in the primers. An additional SV40 NLS (CCAAAGAAGAAGCGGAAGGTC) was included in the reverse primer. The MCP amplicon was inserted in pDONR221 using Gateway BP Clonase II enzyme mix to generate an entry clone. The MCP-NLS ORF was shuttled in pDEST-pcDNA5-BirA*-FLAG_Cterm^25^ using the Gateway LR Clonase II enzyme mix to generate the pDEST-MCP-BirA*-FLAG vector.

To systematically tag lncRNAs with 12 tandem MS2 stemloops at their 3′ end, we built a pUC19-based cloning backbone containing an EF1α core promoter (EFS) and a 12X MS2 tag followed by bGH polyadenylation signal. A bacterial negative selection cassette containing a chloramphenicol resistance gene and the ccdB toxin flanked by BsaI sites with unique overhangs was inserted in between EFS and the MS2 tag to allow Golden Gate-based cloning of lncRNAs of interest. Amplified EFS, ccdB-CmR, 12X MS2-pA, and PGK-Puro-pA fragments were assembled using the In-Fusion HD cloning system according to the manufacturer’s instructions. Plasmids were transformed in One Shot ccdB Survival T21R competent cells which are insensitive to the ccdB negative selection.

LncRNAs were amplified using primer sequences that contained BsaI sites at their 5′ end to allow cloning in the above described backbone. SAMMSON, rcSAMMSON, HOTAIR, and rcHOTAIR were amplified from pLenti-PGK-SAMMSON^26^ or LZRS-HOTAIR (Addgene #26110), respectively, with either Pfu (Agilent 600250) or Q5 (NEB M0491S) polymerase using the following primers with BsaI sites highlighted in italics:

SAMMSON_Fwd: TGAAGCTT*GGTCTC*AACAGGGTGAGGACAGGCGCTCCTGC,
SAMMSON_Rev: CGAGAATTC*GGTCTC*ACGGGGTCCTAGAACTTAAAGTATA,
rcSAMMSON_Fwd: TGAAGCTT*GGTCTC*AACAGGGTCCTAGAACTTAAAGTATA,
rcSAMMSON_Rev: CGAGAATTC*GGTCTC*ACGGGGTGAGGACAGGCGCTCCTGC,
HOTAIR_Fwd: TGAAGCTT*GGTCTC*AACAGGGACTCGCCTGTGCTCTGGAG,
HOTAIR_Rev: CGAGAATTC*GGTCTC*ACGGGTTTGAAAATGCATCCAGATA,
rcHOTAIR_Fwd: TGAAGCTT*GGTCTC*AACAGGTTTGAAATGCATCCAGATA,
rcHOTAIR_Rev: CGAGAATTC*GGTCTC*ACGGGGACTCGCCTGTGCTCTGGAG.

150 ng of each amplicon was mixed with 50 ng pEFS-ccdB-12X MS2, 10 U of BsaI-HFv2 (NEB R3733), and 1X CutSmart buffer for 1 h at 37 °C followed by 20 min at 80 °C to stop the reaction. 1X T4 ligase buffer and 1 U T4 DNA ligase (ThermoFisher Scientific 15224025) were spiked in the digested reaction mixture and left overnight at room temperature. Plasmids were chemically transformed and grown on LB agar plates containing 50 µg/mL carbenicillin. Colonies were verified by restriction digest and Sanger sequencing. The pEFS-ccdB-12X MS2 cloning backbone is made available through Addgene (#177809).

### RNA-BioID

We adapted the RNA-BioID protocol^15^ to our own BioID protocol as applied for bait proteins described in Vandemoortele et al.^27^ and Masschaele et al.^18^. In brief: for each condition, 20.1 × 10^6^ T-REx 293 RNA-BioID cells were plated in triplicate. Next day, cells were transfected with 17.4 µg of the corresponding lncRNA-12X MS2 constructs using PEI. Twenty-four hours post-transfection, the culture medium was refreshed and 2 µg/mL puromycin and 2 ng/mL doxycycline were supplemented to the fresh medium. Forty-eight hours post-transfection 50 µM biotin was added to perform biotin labeling for 16–18 h. Three hours before harvesting, the culture medium was refreshed with biotin-free medium to prevent saturation of the beads by free biotin still present during the enrichment protocol. Cells were washed twice on the plate with 10 mL ice-cold PBS, and were ultimately collected by scraping in 750 µL ice-cold PBS. Cells were pelleted by centrifugation at 500×*g* for 5 min at 4 °C and were washed once more with 10 mL PBS. Cells pellets were resuspended in 5 mL RIPA lysis buffer (50 mM Tris–HCl pH 8.0, 150 mM NaCl, 1% NP-40, 1 mM EDTA, 1 mM EGTA, 0.1% SDS, supplemented fresh with cOmplete™ protease inhibitor cocktail (Roche 11697498001) and 0.5% sodium deoxycholate) and were incubated for 15 min on ice to allow efficient lysis. To each lysate 250 U benzonase was added and incubated with by end-over-end rotation for 1 h at 4 °C. Lysates were subsequently cleared of cellular debris by spinning at 15,000×*g* for 15 min at 4 °C. The supernatant was transferred to a fresh tube and protein concentration of the lysates was determined using the Bradford assay. A maximal shared protein amount across all samples was calculated to ensure equal starting protein material for each sample. Ninety microliters of Streptavidin Sepharose High Performance beads (GE Healthcare GE17-5113-01) per sample were equilibrated by washing three times with 1 mL unsupplemented RIPA buffer, and were eventually resuspended in 90 µL of supplemented RIPA buffer per sample. Cleared lysates were incubated with equilibrated beads by end-over-end rotation for 3 h at 4 °C to enrich for biotinylated proteins. After affinity purification, beads were pelleted by centrifugation at 500×*g* for 2 min. Beads were washed three times with unsupplemented RIPA buffer, twice with 1 mL 50 mM ammonium bicarbonate pH 8.0, and three times with 1 mL trypsin digest buffer (20 mM Tris–HCl pH 8.0, 2 mM CaCl_2_). Beads were ultimately resuspended in 20 µL 20 mM Tris–HCl pH 8.0, and 1 µg trypsin was added and samples were incubated overnight at 37 °C to allow on-bead protein digestion. Next day, another 500 ng of trypsin was added and samples were incubated for 3 h at 37 °C, after which beads were pelleted and supernatant was transferred to a MS vial. Peptide samples were acidified to a final concentration of 2% formic acid. Quality control samples (input, flow through, and enriched fractions) to assess enrichment of biotinylated proteins on Western Blot.

### ChIRP-MS

Eight biotinylated probes complementary to HOTAIR were designed and ordered using the online ChIRP Probe Designer tool available at the BioSearch Technologies website. A probe pool targeting LacZ was designed as a negative control pool. Standard settings were kept as recommended by the manufacturer. HOTAIR probe sequences are:

HOTAIR_1: CAGGACCTTTCTGATTGAGA
HOTAIR_2: TGGTGTAATTGCTGGTTTA
HOTAIR_3: ATCAATTAATTAGCGCCTCC
HOTAIR_4: CAAGTAGCAGGGAAAGGCTT
HOTAIR_5: TGCCAGTTAGAAAAGCGGTG
HOTAIR_6: GGGGTCTATATTTAGAGTGC
HOTAIR_7: AGGAGGAAGTTCAGGCATTG
HOTAIR_8: CCTGAGTCTATTTAGCTACA

Three replicates of 120 × 10^6^ MCF7 cells were cultured per probe pool. The day after, medium was aspirated and cells were washed using 10 mL ice-cold PBS on the plate. Subsequently 10 mL ice-cold PBS was added and RNA–protein interactions were UV crosslinked using a Strategene Crosslinker at 254 nm up to an accumulating energy of 400 mJ/cm^2^. Cells were subsequently scraped and washed once more with 10 mL PBS. Cell pellets were resuspended in 2 mL ChIRP lysis buffer (20 mM Tris–HCl pH 7.5, 200 mM NaCl, 2.5 mM MgCl2, 0.05% NP-40, 0.1% SDS, supplemented fresh with 0.1% sodium deoxycholate, 60 U/mL SUPERase-In RNase inhibitor, 1 mM DTT, 0.5 mM PMSF, and cOmplete protease inhibitor cocktail), and incubated on ice for 15 min to allow efficient lysis. Lysates were sonicated using a Diagenode Bioruptor sonicator using 30 s ON and 45 s OFF per cycle at 4 °C until genomic DNA was fragmented in 500 bp fragments. Proper fragment size was assessed by running 0.5% of the samples on a 1% agarose gel. Sonicated cell lysates were centrifuged at 16,100×*g* for 15 min at 4 °C and supernatant was transferred to a fresh tube. One hundred microliters of Dynabeads MyOne Streptavidin C1 (ThermoFisher 65001) per replicate were rendered RNase-free as by the manufacturer’s instructions, and equilibrated by washing three times with unsupplemented ChIRP lysis buffer. Beads were ultimately resuspended in 100 µL supplemented ChIRP lysis buffer per sample, and 625 pmol of either HOTAIR or LacZ probe pool was added. Beads were then incubated overnight at 4 °C with end-over-end rotation. Next day, lysates were pre-cleared using 30 µL equilibrated beads per sample by end-over-end incubation for 30 min at 4 °C. Pre-cleared lysates were subsequently incubated with 100 µL probe-bound beads for 3 h at 4 °C with end-over-end rotation. After capture, beads were washed three times with 1 mL unsupplemented ChIRP lysis buffer, three times with 1 mL trypsin digest buffer, and finally dissolved in 20 µL 20 mM Tris–HCl pH 8.0. Beads were incubated with 1 µg trypsin overnight at 37 °C. Next day, 500 ng additional trypsin was added and samples were incubated another 3 h at 37 °C. Beads were magnetized and the supernatants containing the peptide mixtures were transferred to an MS-vial and acidified to a final concentration 2% formic acid. Quality control samples (1% input and 10% of the enriched fraction) for all replicates of both probe pools were taken to ensure HOTAIR enrichment in the HOTAIR probe pool replicates. RNA was isolated using QIAzol-chloroform extraction.

### LC–MS/MS

Peptide mixtures were run on a 50 cm µPAC (PharmaFluidics) column connected to a Q-Exactive HF mass spectrometer. The mass spectrometer was operated in a data-dependent acquisition, positive ionization mode, automatically switching between MS and MS/MS acquisition for the five most abundant peaks.

Xcalibur raw files were analysed using the Andromeda search engine as implemented in MaxQuant (v1.6.0.1). Identified spectra were searched against the human proteome (UniProt). Methionine oxidation and N-terminal acetylation were set as variable modifications during the search. Fast LFQ was disabled, and the minimum LFQ ratio was set at 2. LFQ intensities for identified proteins were imported in Perseus (v1.5.8.5) for downstream analysis. In brief, LFQ intensities were log2 transformed and filtered based on reverse hits, contaminant proteins, and proteins identified by site. At least three valid values were needed for a protein to be retained in the analysis. Missing values were imputed from a normal distribution. Finally, a two-sided *t* test (FDR 0.05, 1000 randomizations) was performed to find differential proteins as visualized by volcano plots. Default S0 values were kept during the analysis. Proteomics data sets have been deposited to the ProteomeXchange Consortium through the PRIDE repository (identifiers PXD029057 and PXD029058).

### RNA immunoprecipitation

7.7 × 10^6^ T-REx 293 RNA-BioID cells were transfected with 5.8 µg of lncRNA-12X MS2 construct and 5.8 µg pSV-SPORT using PEI. Twenty-four hours post-transfection 2 µg/mL puromycin and 1 µg/mL doxycycline were added. Next day, cells were washed one on the plate with 5 mL PBS and were collected by scraping in 1 mL PBS. Cell pellets were resuspended in polysome lysis buffer (PLB; 20 mM Tris pH 8.0, 200 mM NaCl, 2.5 mM MgCl_2_, 0.05% Triton X-100) and incubated on ice for 15 min to promote lysis. Lysates were centrifuged at 15,000×*g* for 5 min at 4 °C and the supernatant was transferred to a fresh tube. 10 µg of FLAG M2 antibody (Sigma Aldrich F3165-1MG) or 10 µg of IgG isotype control (Abcam ab18443) was conjugated to 100 µL of Dynabeads Protein G (ThermoFisher Scientific 10004D) in 1 mL Tris wash buffer (20 mM Tris–HCl pH 7.5, 150 mM NaCl) for 30 min at room temperature. After conjugation, beads were washed twice with 1 mL PLB and ultimately resuspended in 100 µL PLB. Lysates were incubated with antibody-conjugated beads for 3 h at 4 °C with end-over-end rotation. Beads were washed three times with 1 mL PLB. Captured RNA transcripts were eluted from the beads by adding 95 µL proteinase K digestion buffer (10 mM Tris–HCl pH 7.5, 100 mM NaCl, 1 mM EDTA, 0.5% SDS) supplemented with 5 µL proteinase K. The reaction was incubated at 50 °C for 45 min, followed by 10 min at 95 °C. Samples were cooled down to room temperature and 500 µL QIAzol was added. After a 10 min incubation time at room temperature, 100 µL chloroform was added and samples were vigourously vortexed and centrifuged at 16,000×*g* for 15 min at 4 °C. The upper aqueous phase was transferred to a fresh tube. For each 200 µL RNA, a 1:1 mix of 300 µL RA1 (Macherey-Nagel 740961) and 300 µL 100% EtOH was added. RNA from the mixture was isolated using the Nucleospin RNA mini kit (Macherey-Nagel 740955.250) according the manufacturer’s instructions. Input, flow through, and enriched fractions were isolated during enrichment protocol both for Western Blot and RNA isolation.

### cDNA synthesis and RT-qPCR

cDNA synthesis was performed using the PrimeScript RT kit (Takara Bio RR037A) using maximal shared RNA input across samples for RIP experiments or maximal volume for ChIRP experiments. Target transcripts were amplified using primers listed below using the SensiFAST SYBR No-ROX kit (Meridian Bioscience BIO-98005) and signal was detected using a LightCycler 480.

SDHA_Fwd: TGGGAACAAGAGGGCATCTG
SDHA_Rev: CCACCACTGCATCAAATTCATG
YWHAZ_Fwd: ACTTTGGTACATTGTGGCTTCAA
YWHAZ_Rev: CCGCCAGGACAAACCAGTAT
UBC_Fwd: ATTTGGGTCGCGGTTCTTG
UBC_Rev: TGCCTTGACATTCTCGATGGT
HOTAIR_Fwd: GGTAGAAAAAGCAACCACGAAGC
HOTAIR_Rev: ACATAAACCTCTGTCTGTGAGTGCC
SAMMSON_Fwd: CCTCTAGATGTGTAAGGGTAGT
SAMMSON_Rev: TTGAGTTGCATAGTTGAGGAA

Samples were measured in technical quadruplicates in a 384-well plate as described by the manufacturer’s instructions. The following cycling conditions were used: 1 cycle at 95 °C for 5 min, 40 cycles at 95 °C for 10 s, 60 °C for 10 s and 72 °C for 10 s, followed by melting curve analysis to validate unique amplicons. Quantitation cycles (Cq) were normalized to housekeeping genes (SDHA, UBC, YWHAZ) using geometric averaging^28^. The geNorm algorithm was used to calculate the stability of the housekeeping genes. Subsequently, the normalized Cqs were compared relative to the corresponding input sample. All RT-qPCR analyses were done in qbase+.

### SDS-PAGE and western blot

For input and depleted samples, 30 µg of protein material was measured using Bradford reagent (Bio-Rad Protein Assay Dye Reagent concentrate #5000006). For enriched samples, 5% of the sample was used. To each sample, 7.5 µL XT Sample Buffer (Bio-Rad #1610791) and 1.5 µL XT Reducing Agent (Bio-Rad #1610792) was added and supplemented with water to a final volume of 30 µL. Samples were heated to 95 °C for 10 min. Samples were first cooled down to room temperature, loaded and ran on a 4–12% ExpressPlus PAGE 4–12% pre-cast gel (Genscript M421215) according to the manufacturer’s instructions. Proteins were transferred to PVDF membrane (Merck #IPFL00010) for 3 h at 60 V in Disolol blotting buffer (Chem-lab CL00.1807.5000). Membranes were blocked for 30 min at room temperature by incubation with Odyssey Blocking buffer (LI-COR 927-50000). Primary antibodies were incubated overnight at 4 °C with gentle end-to-end rotation. The following primary antibodies were used: rabbit polyclonal anti-ACTB (Sigma #A2066) at 1/2000 and mouse monoclonal anti-FLAG M2 (Sigma #F1804) at 1/1000 in TBS. Membranes were washed three times with TBS-Tween 0.1% (v/v) for 10 min at room temperature with gentle end-to-end rotation. Secondary antibodies were incubated for 1 h at room temperature. The following secondary antibodies were used: goat polyclonal anti-mouse IgG IRDye 800CW (LI-COR), goat polyclonal anti-rabbit IgG 800CW (LI-COR), goat polyclonal anti-mouse IgG IRDye 680RD (LI-COR), and goat polyclonal anti-rabbit 680RD (LI-COR). All secondary antibodies were used at a 1/5000 dilution. After secondary antibody incubation, membranes were washed again as described previously and visualized on a LI-COR Odyssey IR scanner. For RNA-BioID experiments, after visualization, membranes were incubated for 1 h at room temperature with IRDye 680RD Streptavidin (LI-COR) to visualize biotinylation. Membranes were washed once more before visualization.

### RNAscope and MitoTracker staining

Prior to seeding cells, each chamber of a 8-well glass chamber slide was incubated for 20 min at room temperature with 2.8 µg Cell-Tak (Corning 354240) dissolved in 100 µL filter-sterilized 0.1 M NaHCO_3_ pH 8.0 to coat each chamber. After washes, 10,000 MCF7 cells were seeded in each chamber in serum-free growth medium to stimulate adhesion. After attachment, medium was refreshed to complete growth medium and cells were grown to a confluency of 50–75%.

One microliter MitoTracker Orange CMTMRos (ThermoFisher Scientific M7510) was diluted in 10 mL serum-free and additive-free growth medium before use. Growth medium of the cells was removed and cells were incubated with MitoTracker Orange CMTMRos containing growth medium for 30 min on 37 °C. Immediately afterwards, slides were fixed with 4% PFA for 30 min at room temperature and stored in the dark until further processing. RNAscope for HOTAIR (Advanced Cell Diagnostics cat. 312341) was performed according to the manufacturer’s instructions using the RNAscope Multiplex Fluorescent V2 Assay (Advanced Cell Diagnostics cat. 323100) for adherent cells. Control probes in the experiment as advised by the manufacturer were *B. subtilis* DapB (Advanced Cell Diagnostics cat. 310043) as a negative control and *H. sapiens* PPIB (Advanced Cell Diagnostics cat. 313901) as a medium expressed universal positive control. DAPI stained was performed to stain nuclei. Slides were analysed with a confocal laser scanning microscope. To overcome cross-excitation, the relative contribution of each fluorophore for every pixel was determined by linear unmixing. Colocalization was determined by intersect analysis of both fluorophores. Absolute counts of HOTAIR in each cellular compartment were determined by simply counting the number the dots present in each compartment.

## Results

### A straightforward assembly method to tag lncRNAs with MS2 stem loops

To easily tag lncRNAs with MS2 stem loops, we generated an assembly vector (Fig. S1) that expresses lncRNAs from an EF1α core promoter (EFS) with a 12X tandem MS2 stem loop at the 3′ end. To perform scarless cloning of the lncRNA, we took advantage of the type IIS restriction enzyme BsaI that is frequently used in Golden Gate assembly methods. We inserted BsaI recognition sites directly downstream of the EFS sequence and upstream of the 12X MS2 tag that would eliminate themselves during the cloning process. In between both BsaI sites, we inserted a *ccdB* negative selection cassette to allow for a one-pot assembly of the lncRNA within the vector, effectively eliminating the need to gel purify BsaI-cut vector and insert. As the 5′ BsaI site downstream of the EFS promoter cuts within the EFS promoter itself, we reconstituted the 5′ end of the promoter sequence within the primer sequence that we used to amplify the lncRNA. An additional puromycin resistance expression cassette was inserted in the vector to perform selection of positively transfected clones. We efficiently cloned the lncRNAs HOTAIR and SAMMSON, as well as their reverse complements (rcHOTAIR and rcSAMMSON) scarlessly within this assembly vector using BsaI overhangs within the primer sequences.

### Purification of HOTAIR-interacting proteins by ChIRP-MS

**Figure 1:**
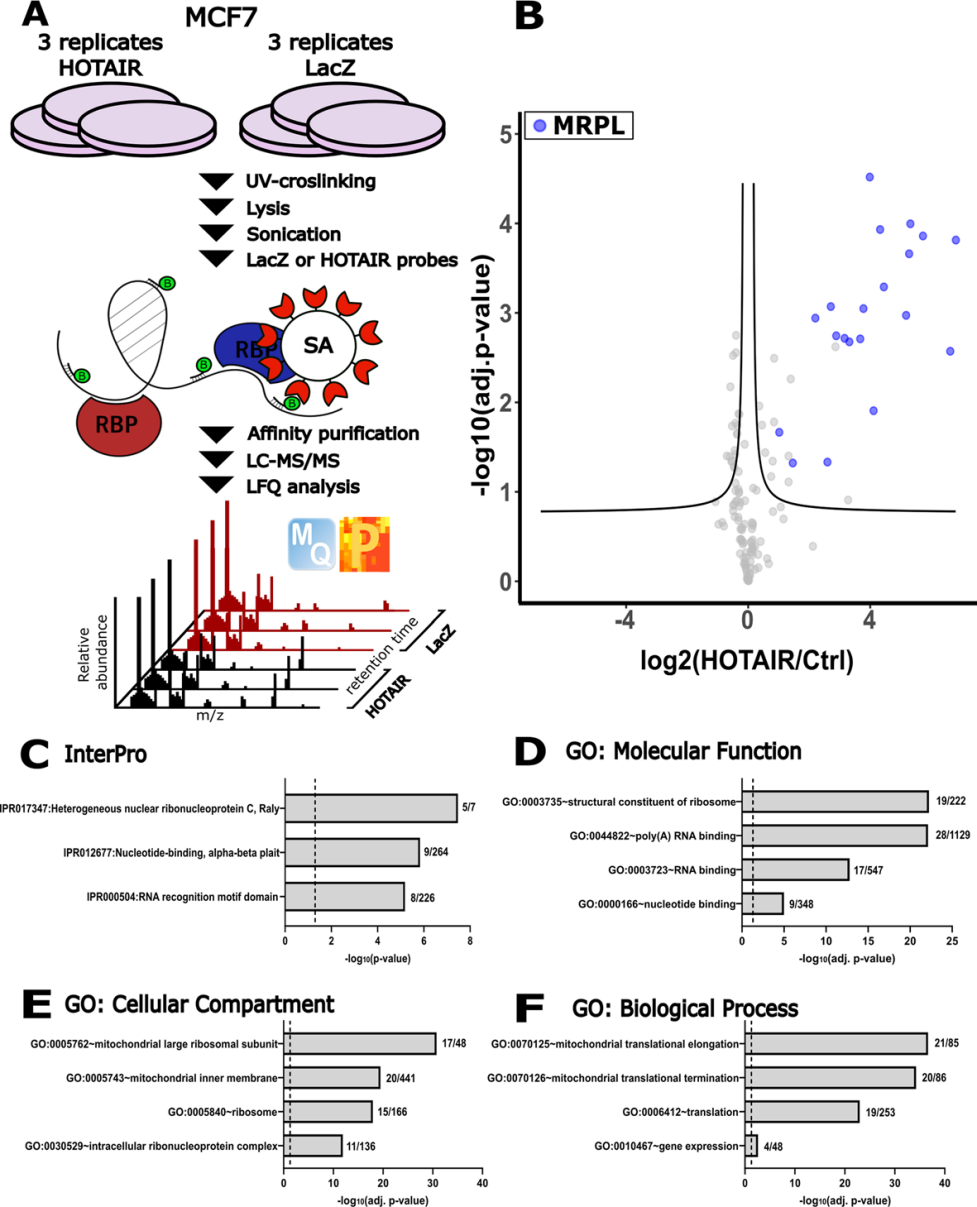
Identification of HOTAIR-interacting proteins by ChIRP-MS. (**A**) Schematic overview of ChIRP-MS. (**B**) Volcano plot showing the potential protein interactors of HOTAIR by ChIRP-MS (FDR 0.05; s0 0.1). MRPLs are highlighted in blue. InterPro domains overrepresented in the data set (**C**). Gene Ontology analysis showing Molecular Function (**D**), Cellular Component (**E**), and Biological Process (**F**). GO was performed with David 6.8., the four most significant terms are shown. The vertical dashed line represents the 0.05 cutoff used for the adjusted p-value. Number of significant proteins identified and the size of each annotation is shown next to each histogram. *SA* streptavidin, *RBP* RNA-binding protein, *LFQ* label free quantification.

To identify HOTAIR-interacting proteins in an unbiased way in whole cell lysates, we applied ChIRP-MS (Fig. 1A) on the endogenous HOTAIR in MCF7 breast cancer cells, a commonly used cell line in HOTAIR research. Before advancing with LC–MS/MS analysis, we confirmed enrichment of the HOTAIR transcript compared to the LacZ negative control probe pool (Fig. S2A). Pearson correlation showed replicates correlated well between conditions (Fig. S2B). We identified 33 proteins that were significantly (FDR 0.05, Table S1) enriched in the HOTAIR pulldown (Fig. 1B). Proteins containing RNA binding domains (hnRNP, RRM) were significantly overrepresented (Fig. 1C) in the enriched proteins showing the robustness of ChIRP-MS to enrich for RNA-binding proteins. GO molecular function analysis also indicated that RNA binding was one of the main annotated functions among the enriched proteins (Fig. 1D). Surprisingly, we did not identify any MS/MS spectra of the known HOTAIR-interacting proteins in any of the HOTAIR or control samples. However, 21 out of 33 identified proteins that were significantly enriched in the HOTAIR samples were mitoribosomal proteins (MRPLs, Fig. S2C) that are part of the 39S large mitoribosomal (Fig. 1E,F). In addition, we identified HSP10, LRPPRC, and GTPBP6, all of which have previously been implicated in mitochondrial import, mitochondrial translation, or both.

### Proximity labeling of HOTAIR-interacting proteins by RNA-BioID

**Figure 2:**
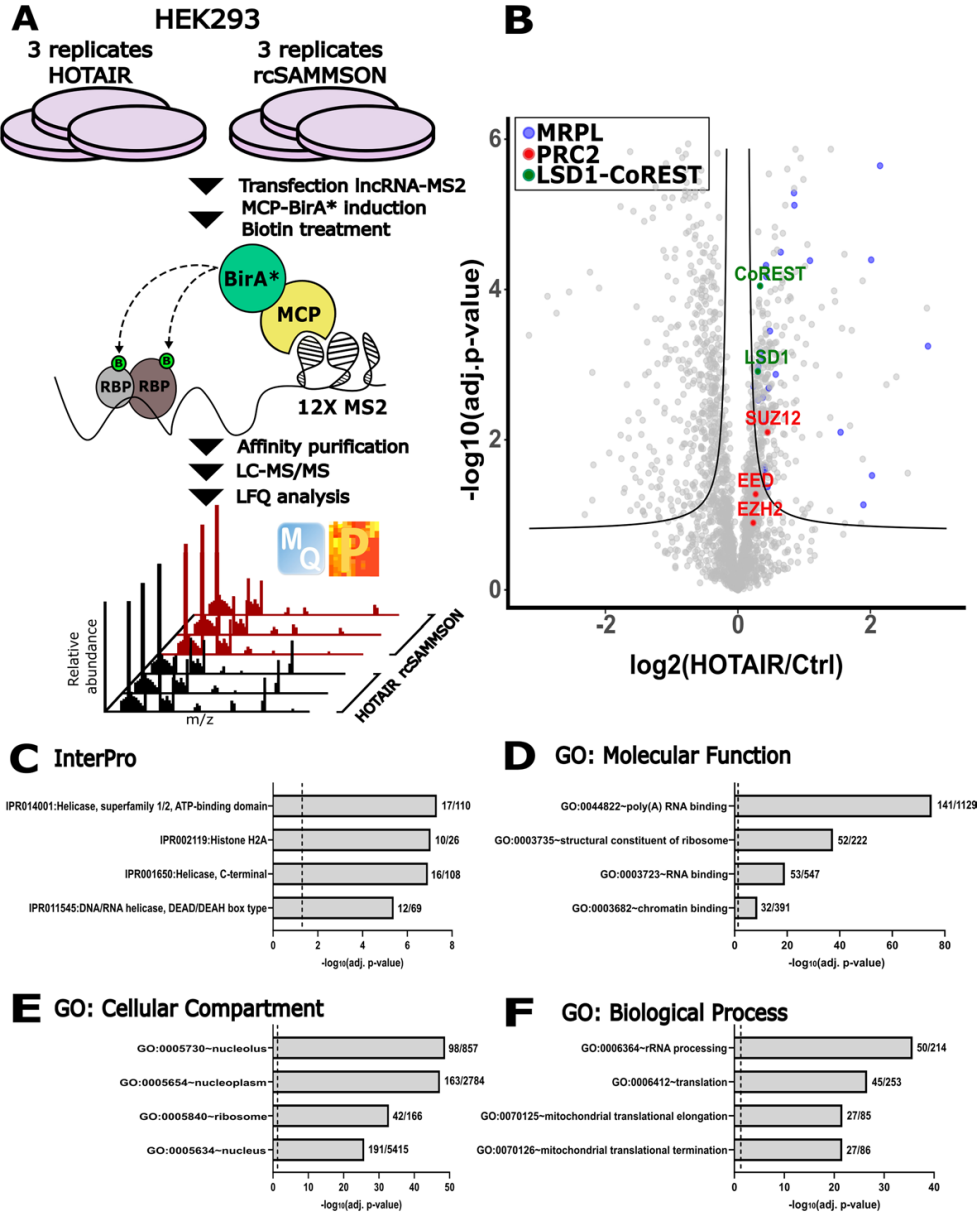
Identification of HOTAIR-interacting proteins by RNA-BioID. (**A**) Schematic overview of RNA-BioID. (**B**) Volcano plot showing the potential protein interactors of HOTAIR by RNA-BioID (FDR 0.05; s0 0.1). MRPLs are highlighted in blue, PRC2 complex members are highlighted in red, LSD1-CoREST members are highlighted in green. InterPro domains overrepresented in the data set (**C**). GO analysis showing Molecular Function (**D**), Cellular Component (**E**), and Biological Process (**F**). GO was performed with David 6.8., the four most significant terms are shown. The vertical dashed line represents the 0.05 cutoff used for the adjusted p-value. Number of significant proteins identified and the size of each annotation is shown next to each histogram. *MCP* MS2 coat protein, *RBP* RNA-binding domain, *LFQ* label free quantification.

To better understand our ChIRP-MS results, we opted to also perform RNA-BioID (Fig. 2A), an orthogonal proteomic method to find HOTAIR-interacting proteins. Therefore, we integrated a tandem MS2 coat protein dimer fused to the promiscuous biotin ligase BirA* (MCP-BirA*) in the Flp-In T-REx 293 cell line, derived from 293 embryonic kidney cells, to regulate the expression of MCP-BirA* using doxycycline. Constructs expressing HOTAIR, or control plasmids expressing either rcHOTAIR or rcSAMMSON, with an 12X MS2 tag at the 3′ were transiently transfected. We confirmed binding of the MCP-BirA* protein to the MS2-tagged lncRNAs by performing RIPs with the C-terminal FLAG-tag (Fig. S3) and biotinylation of the LC–MS/MS samples was confirmed on WB (Fig. S4) before proceeding. Originally, we used both rcHOTAIR and rcSAMMSON control conditions together to identify differential proteins. When doing so, however, we were unable to find any significantly differential proteins (Fig. S5A). However, we argued that the reverse complement of HOTAIR might act as a high-affinity trap for binding the, albeit lowly expressed, endogenous HOTAIR transcripts. Recently, Balas et al.^29^ showed that in vitro titration of HOTAIR with its reverse complement promoted PRC2 activity. This suggests that the reverse complement of a lncRNA might be able to bind its endogenously expressed counterpart, and as such bias the results. Therefore, we decided to only include the rcSAMMSON condition as a scrambled-like control, as SAMMSON itself is a melanoma-specific lncRNA which is not expressed in HEK293 cells. The reverse complement of SAMMSON will therefore not introduce bias in the analysis. Replicates between conditions correlated well as shown by Pearson correlation, however the analysis also demonstrated that a considerable part of the identified proteins were also identified in the rcSAMMSON replicates indicating a sizeable shared background (Fig. S5B,C), which is expected for BioID experiments. We identified 317 significantly enriched proteins (FDR 0.05, Table S2), including previously published interactors such as PRC2 complex component SUZ12 and REST/CoREST components LSD1 and CoREST (Fig. 2B), demonstrating the validity of RNA-BioID. Identified proteins were significantly overrepresented for helicase domains (Fig. 2C) as well as RNA and chromatin binding functions (Fig. 2D) consistent with HOTAIR’s function as an epigenetic regulator. Indeed, most proteins seemed to be associated with the nucleus as shown by GO analysis of the enriched proteins (Fig. 2E). As BioID is a proximity labeling method, we looked also for other members of the PRC2 complex in our data set, both EED and EZH2 were identified and trending towards the HOTAIR-interacting proteins, however they did not pass the significance threshold. GO analysis also showed an overrepresentation of proteins associated with various aspects of mitochondrial translation (Fig. 2F). Indeed, similar as in ChIRP-MS, we identified 14 MRPLs (MRPL3, MRPL9, MRPL11, MRPL12, MRPL17, MRPL18, MRPL19, MRPL22, MRPL23, MRPL27, MRPL28, MRPL38, MRPL41, and MRPL47) to be significantly enriched in the HOTAIR samples.

### The HOTAIR-MRPL interaction does not occur in mitochondria

**Figure 3:**
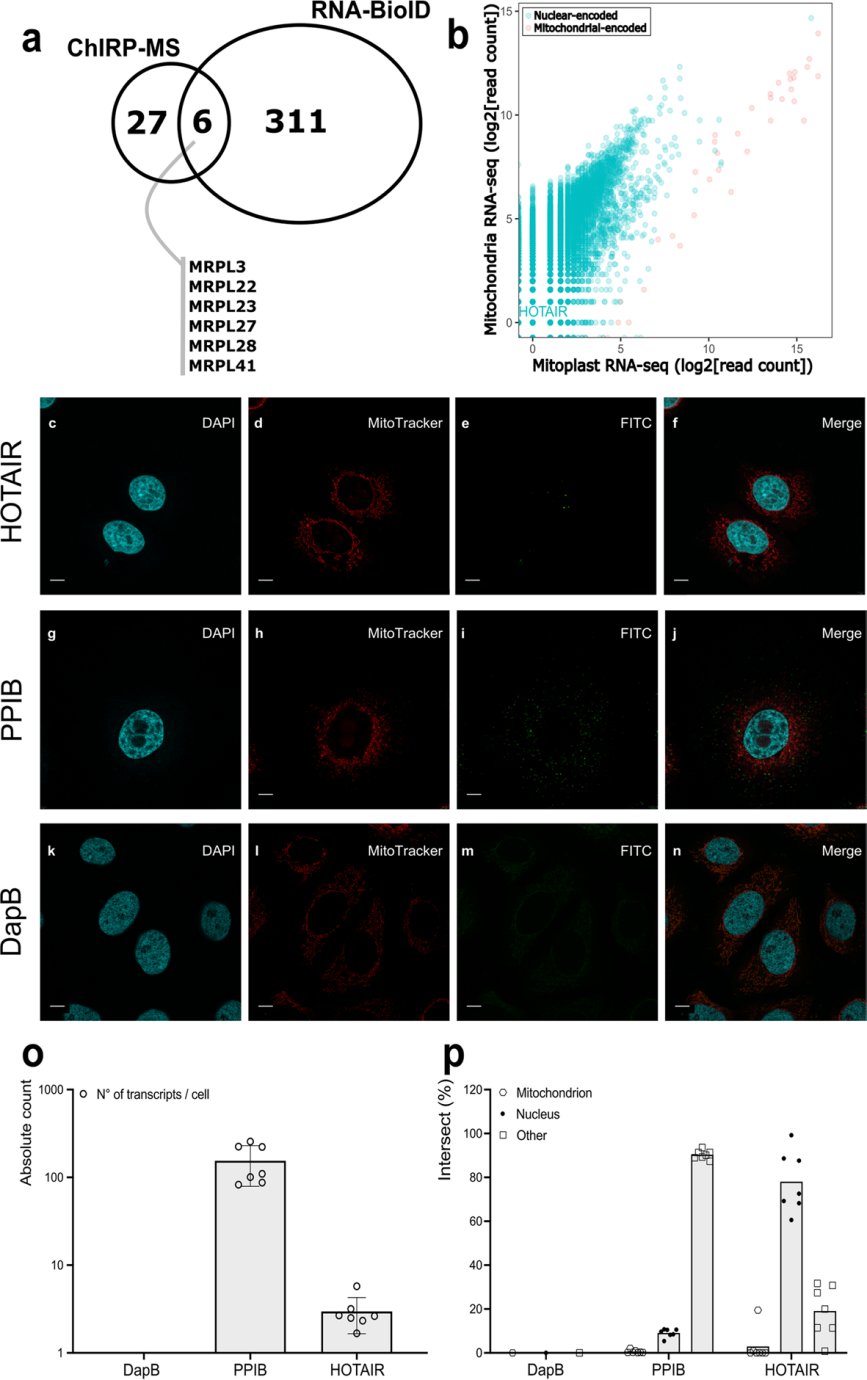
HOTAIR-MRPL interactions do not occur in mitochondria. (**A**) Overlap of ChIRP-MS and RNA-BioID identified proteins. Proteins identified in both methods are shown. (**B**) Reanalysis of RNA-sequencing data of Mercer et al. Nuclear-encoded genes are shown in green. Mitochondrial-encoded genes are shown in red. HOTAIR is highlighted. Colocalization of HOTAIR (**C**–**F**), PPIB mRNA positive control (**G**–**J**), and DapB mRNA negative control (**K**–**N**) with mitochondria in MCF7 determined by staining with RNAscope (FITC) and MitoTracker. (**O**) Determined transcript abundances per cell (n = 7) for each of the targets based on RNAscope puncti. (**P**) Intersect analysis (n = 7) showing percentage of the total transcript pool with mitochondria (hexagons) or nuclei (circles). Transcripts present in unstained organelle are shown as other (squares). The scale bar depicts 7.5 µm.

When overlapping the differential proteins of both orthogonal proteomic methods, we found that 6 proteins were shared between both data sets (Fig. 3A). All 6 proteins were MRPLs, suggesting HOTAIR might bind these proteins to perform functions independently of its transcriptional mode-of-action. MRPLs are the main protein constituents of the 39S subunit of the mitoribosome. Therefore, we wondered whether it could be possible that HOTAIR is imported within mitochondria to be translated by the mitoribosome. We reanalyzed public mass spectrometry data sets of the PRIDE repository using Ionbot^30^ to see if we could detect any peptide-spectrum matches (PSMs) of in silico predicted peptides with the mitochondrial genetic code in all frames. The distribution of PSMs for HOTAIR did not differ between target and decoy databases (Fig. S6A), indicating the transcript is not translated using the mitochondrial genetic code. As a positive control, we reran the analysis with MT-CO3, a mitochondrially translated mRNA. Here, as expected, the distribution of PSM to the target database was highly distinct from the decoy distribution correctly indicating that MT-CO3 is translated (Fig. S6B).

As MRPLs are mainly localized to mitochondria, we wondered whether the potential interaction between these MRPLs and HOTAIR also takes place in mitochondria. We reanalyzed RNA-seq data from Mercer et al*.*^31^, where they performed RNA-seq on both full mitochondria (NCBI GEO: GSM763529) and mitoplast RNA (NCBI GEO: GSM763530). We found only one read for HOTAIR in mitochondrial RNA-seq, indicating HOTAIR is not present in mitochondria (Fig. 3B). To corroborate on this for MCF7 specifically, we visualized the localization of HOTAIR in MCF7 cells using RNAscope (Fig. 3C–F). We also stained PPIB mRNA as a positive control which is expressed in most tissues at medium levels (Fig. 3G–J), as well as the bacterial mRNA DapB (Fig. 2K–N) as a universal negative control. We determined that HOTAIR is present at less than 10 transcripts per cell while PPIB is present at roughly 100 transcripts per cell (Fig. 3O), which is consistent with RNA-seq data from the RNA Atlas^32^ (Fig. S7). Our colocalization analysis showed that HOTAIR is mainly present at the nucleus (80%), with no evidence for a mitochondrial localization (Fig. 3P).

## Discussion

In this work, we set out to screen for potential interaction partners of the lncRNA HOTAIR and identified multiple MRPLs to be associated with HOTAIR. Although we did not identify PRC2 complex or REST/CoREST components in our ChIRP-MS screen of endogenous HOTAIR in MCF7 cells, we did find SUZ12, LSD1, and CoREST to be significantly enriched in the RNA-BioID setup, which was performed on overexpressed HOTAIR in HEK293 cells. In addition, although they did not pass the significance threshold, we also identified EED and EZH2 trending towards the HOTAIR-enriched proteins. Although HEK293 cells do not provide a relevant biological background for HOTAIR research, these cells are often used for BioID studies because of their high transfection efficiency and ease-of-use. It is also worth noting that although HOTAIR is not been commonly studied in HEK293, it is endogenously expressed in HEK293T cells to levels similar as those found in MCF7. Interestingly, MCF7, and almost all other breast cancer cell lines, does not express HOTAIR at the high levels seen in patient samples^33^. Indeed, we quantified HOTAIR copy number in MCF7 cells using RNAscope and found that it was present at less than 10 transcripts per cell (Fig. 3O). Although we do not know the number of HOTAIR transcripts per cell, during RNA-BioID, we overexpressed HOTAIR to much higher levels, suggesting that the expression level of HOTAIR might be crucial to detect PRC2 and LSD1 complexes. Li et al.^34^ performed ChIRP-MS to identify HOTAIR-interacting proteins in whole cell lysates of HeLa cells, which express even lower amount of endogenous HOTAIR compared to MCF7. Similarly, they did not identify PRC2 or LSD1 complex subunits and attributed this to highly abundant cytoplasmic proteins masking the LC–MS/MS signal of these known interactors. They also did not identify any MRPLs to be among the HOTAIR interacting proteins. Overlapping their ChIRP data with our data sets shows only 1 overlapping protein (NCL) with our ChIRP data, and 9 overlapping proteins with RNA-BioID (DHX15, RPL12, RPL18, RPL18A, RPL27A, RPL30, RPS21, RPS3A, and UBE2I). Differences in crosslinking might explain this small overlap, which is also reflected in the number of proteins identified. Li et al. used paraformaldehyde (PFA) to crosslink RNA–protein complexes and found 348 proteins to be significantly enriched in their ChIRP-MS data. PFA is known to crosslink both RNA–protein and protein–protein complexes, strongly increasing the number of proteins identified. We on the other hand performed UV crosslinking, which is a ‘zero distance’ crosslinking strategy that only captures direct RNA–protein interactions, which explains the lower number of proteins identified here. Interestingly, although not retained in their data analysis, they also uniquely identify HSP10 (*HSPE1*) in all HOTAIR pulldown replicates while not being identified in their negative control samples. We also find HSP10 to be a HOTAIR-interacting protein in our ChIRP-MS data. HSP10 is a heat shock protein that together with HSP60 forms multichaperonin complex that acts in the import and proper folding of mitochondrial proteins. Wu et al.^35^ analyzed changes in transcriptomic and proteomic profiles after HOTAIR knockdown. Their data shows that HSP10 is upregulated at the protein level after HOTAIR depletion, yet its transcript level remains unchanged. Interestingly, they also find MRPL12, MRPL41, and MRPL49 to be upregulated at the protein level without changes in their transcript abundances. This suggest that HOTAIR might play a role at the post-translational level of these MRPLs by affecting their stability, folding, import or a combination of these processes. As most studies have used nuclear extracts to study the role of HOTAIR in cancer cell lines, it might be interesting to have a look at the role that the cytoplasmic HOTAIR fraction performs in these cell lines. Zheng et al.^36^ demonstrated that silencing of HOTAIR by RNAi induced mitochondrial dysfunction in HeLa cells. Phenotypically, they observed mitochondrial swelling and loss of cristae, as well as a progressive disappearance of mitochondria in general. They associated this with a decrease in membrane potential (ΔΨ). Similarly, Kong et al.^37^ found a change in ΔΨ after HOTAIR depletion in HNSCC cells. These studies highlight that HOTAIR might function in different pathways and different subcellular locations next to its established function as an epigenetic regulator in the nucleus. Indeed, Yoon et al.^38^ showed that HOTAIR can act as a scaffold for the E3 ubiquitin ligases DZIP3 and MEX3B. HOTAIR overexpression promotes the proteolysis of their targets ATXN1 and SNUPN, respectively. Interestingly, Zhang et al.^39^ demonstrated that PRC2 and MEX3B occupancy on HOTAIR is mutually exclusive, which might explain why we did not pick up DZIP3 or MEX3B in our RNA-BioID screen.

We did not find any evidence for a mitochondrial localization of HOTAIR in MCF7 cells, suggesting that HOTAIR binds these MRPLs in another compartment of the cell. As MRPLs are nuclear-encoded and therefore translated in the cytoplasm, it is not unlikely that HOTAIR binds these MRPLs after being translated and before being imported in the mitochondria. Smirnov et al.^40^ suggested that nuclear-encoded 5S rRNA could bind cytosolic pre-MRPL18. The association between both molecules confers a conformational change in the 5S rRNA allowing it to be imported in mitochondria. This shows that MRPLs can interact with nuclear-encoded transcripts as well as interact with them in the cytosol. Recently, although we did not highlight the location of the interaction, we showed that the melanoma-specific lncRNA SAMMSON also interacts with MRPLs in uveal melanoma^41^.

As MRPLs are highly abundant proteins present in the cell, we wondered if these are commonly found in other ChIRP-MS experiments. Flynn et al.^42^ performed ChIRP-MS to screen RNA-host protein interactions with the RNA genomes (vRNAs) of SARS-CoV-2, Dengue virus (DENV), Zika virus (ZIKV), and rhinovirus (RV). They identified specific MRPLs interacting with SARS-CoV-2 viral RNA (vRNA), which were different from the multiple MRPLs that were shown to interact with RV vRNA. No MRPLs were shown to interact with DENV and ZIKV vRNAs.

We believe there to be great merit in performing orthogonal proteomics methods to evaluate the interactome of a lncRNA. While separate interactome studies clearly provide candidate partner lists that are challenging to prioritize, the combination of both orthogonal methods shows a limited list that reveals a surprising association of HOTAIR with MRPLs. These interactions remain to be functionally explored, but our results already highlight that combining orthogonal methods provide a high confidence set of protein interactors, similar as seen in PPI studies^17,19–21^. However, even in the study presented here, biases attributed to differences in cell lines might play an important role. Recently, Yu et al.^43^ showed that the lncRNA Xist has a B-cell-specific interactome with TRIM28 being a unique interactor in female B-cells, which demonstrates that lncRNAs can have cell type and probably also cell line-specific interactomes. Therefore, it would be interesting to see what RNA-BioID approaches in HOTAIR research would reveal when applied to cell lines more relevant than HEK293 cells such as MCF7 cells. Similarly, HOTAIR expression levels can contribute to differences in identified proteins. Therefore, performing a set of ChIRP-MS or RNA-BioID experiments to map changes in the HOTAIR interactome upon varying HOTAIR transcript abundances might be very valuable to evaluate the impact of HOTAIR expression levels on changes of its identified interactome. In addition, the MS2 system (either the tag itself or binding of MCP to the MS2-tag) might displace protein interactors. Therefore, it might be useful to explore both 5′ and 3′ tagging. Optimally, an endogenously MS2-tagged lncRNA would be the most relevant way forward for these kinds of approaches. However, endogenous tagging of non-coding genes remains challenging. Endogenously engineering antisense genes in a locus might influence the expression of the sense gene, which is concerning as lncRNAs have been shown to act in cis. In addition, differences in RNA stability due to the introduction of the tag might change expression levels compared to wild type levels.

In conclusion, we performed two independent methods to identify HOTAIR-interacting proteins. Overlap of both methods revealed an association with mitoribosomal proteins of the large 39S subunit, although this association does not seem to happen within mitochondria. Future research to validate and localize this interaction between MRPLs and HOTAIR is needed to show functionality of this potential interaction.

## Supplementary Information

Supplementary Table S1.

Supplementary Table S2.

Supplementary Table S3.

Supplementary Figures.

## Data Availability

Proteomics data sets have been deposited to the ProteomeXchange Consortium through the PRIDE repository (identifiers PXD029057 and PXD029058). The pEFS-ccdB-12X MS2_mPGK-PuroR assembly backbone is available through Addgene (#177809).
