# Supplementary material for: Orthogonal proteomics methods to unravel the HOTAIR interactome
Source: Sci Rep. 2022 Jan 27;12:1513. doi: 10.1038/s41598-022-05405-6 (PMC8795419; doi:10.1038/s41598-022-05405-6)
Supplement: Supplementary file 4 — Supplementary Figures. [file 41598_2022_5405_MOESM4_ESM.pdf]

Supplementary Figure 1

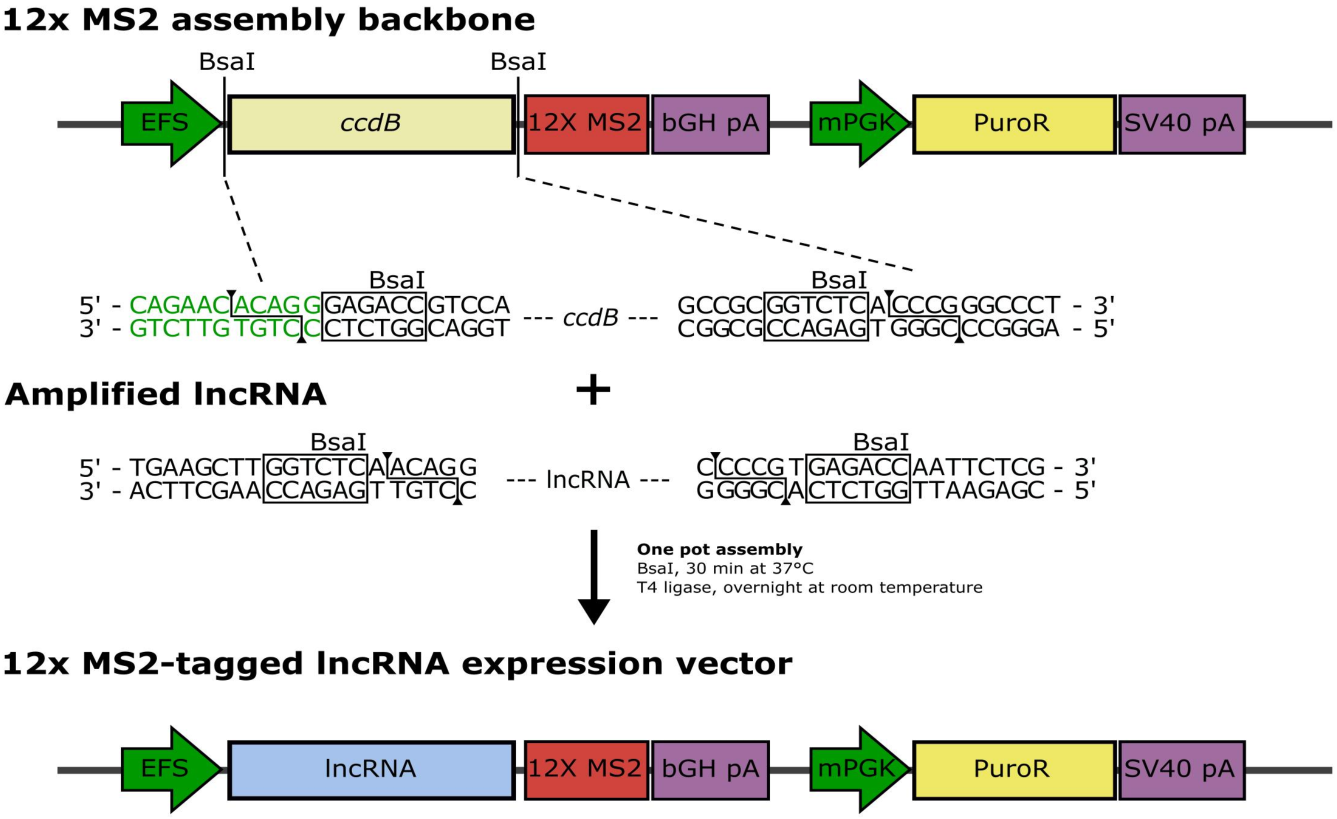

Figure S1: Schematic overview of the cloning strategy to tag lncRNAs with 12X MS2 stemloops. During the one-pot assembly the negative selection cassette of the assembly backbone is switched out for an amplified lncRNA by using unique BsaI overhangs. The EFS promoter sequence is shown in green. EFS, EF1α core promoter; bGH, bovine Growth Hormone polyadenylation signal; mPGK, murine phosphoglycerate kinase promoter; PuroR, human codon-optimized puromycin *N*-acetyl-transferase (*pac*) gene conferring resistance to puromycin in mammalian cell culture; SV40 pA, SV40 early polyadenylation signal; *ccdB*, bacterial toxin to allow negative selection; lncRNA, long non-coding RNA.

Figure S2

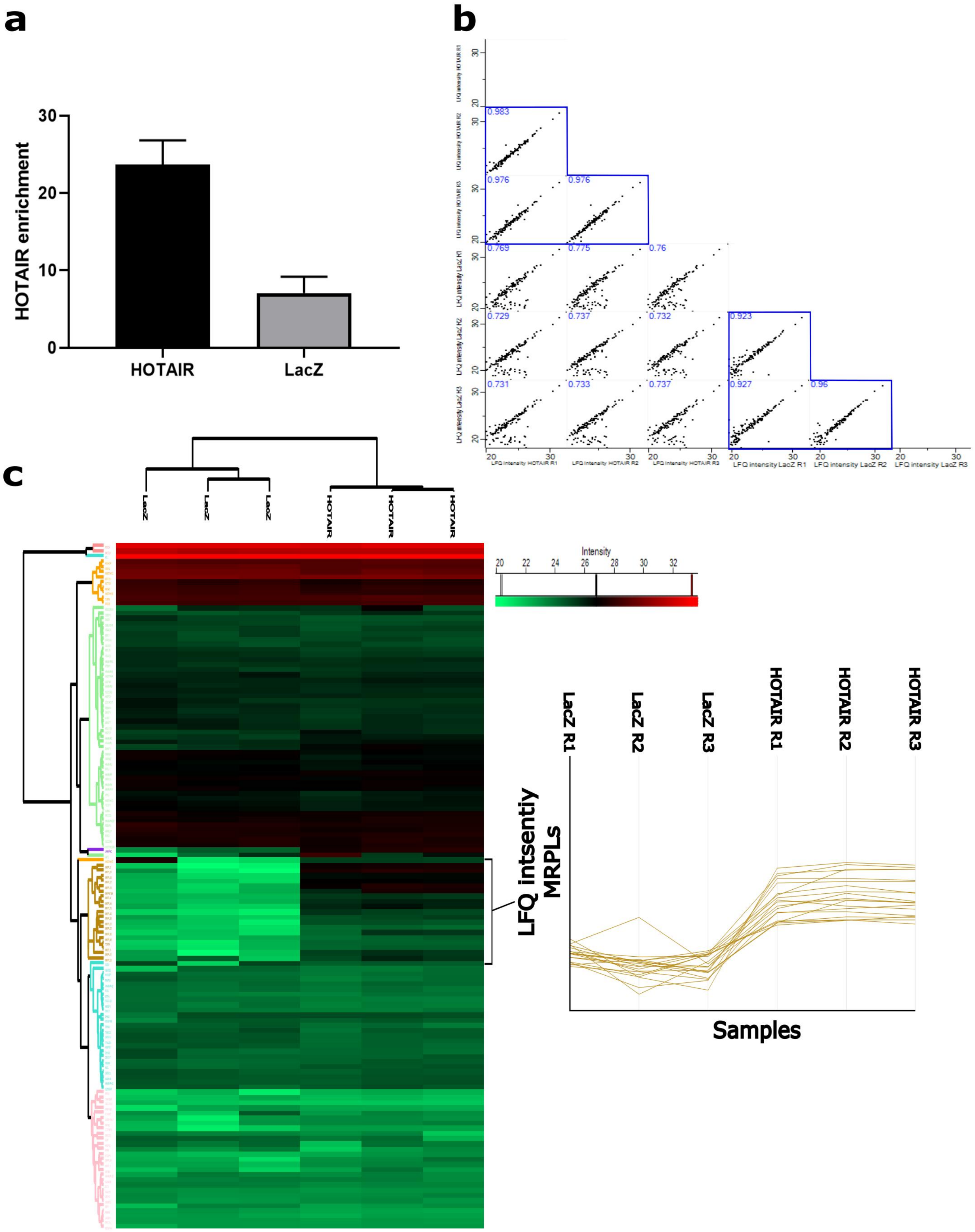

Figure S2: Quality controls for ChIRP-MS samples. **(a)** HOTAIR enrichment in the HOTAIR and LacZ pulldown samples as measured by RT-qPCR. Mean ± SD of all three replicates is shown. **(b)** Scatter plot showing the Pearson correlation coefficient of all replicates. **(c)** Heatmap of all proteins in all samples. Clustering of samples and proteins was done using Euclidean Hierarchical clustering. LFQ intensities of rows and columns were preprocessed with k-means and clustered based on the average-linkage. The profile plot of the LFQ intensities of the MRPL cluster is highlighted on the right.

Supplementary Figure 3

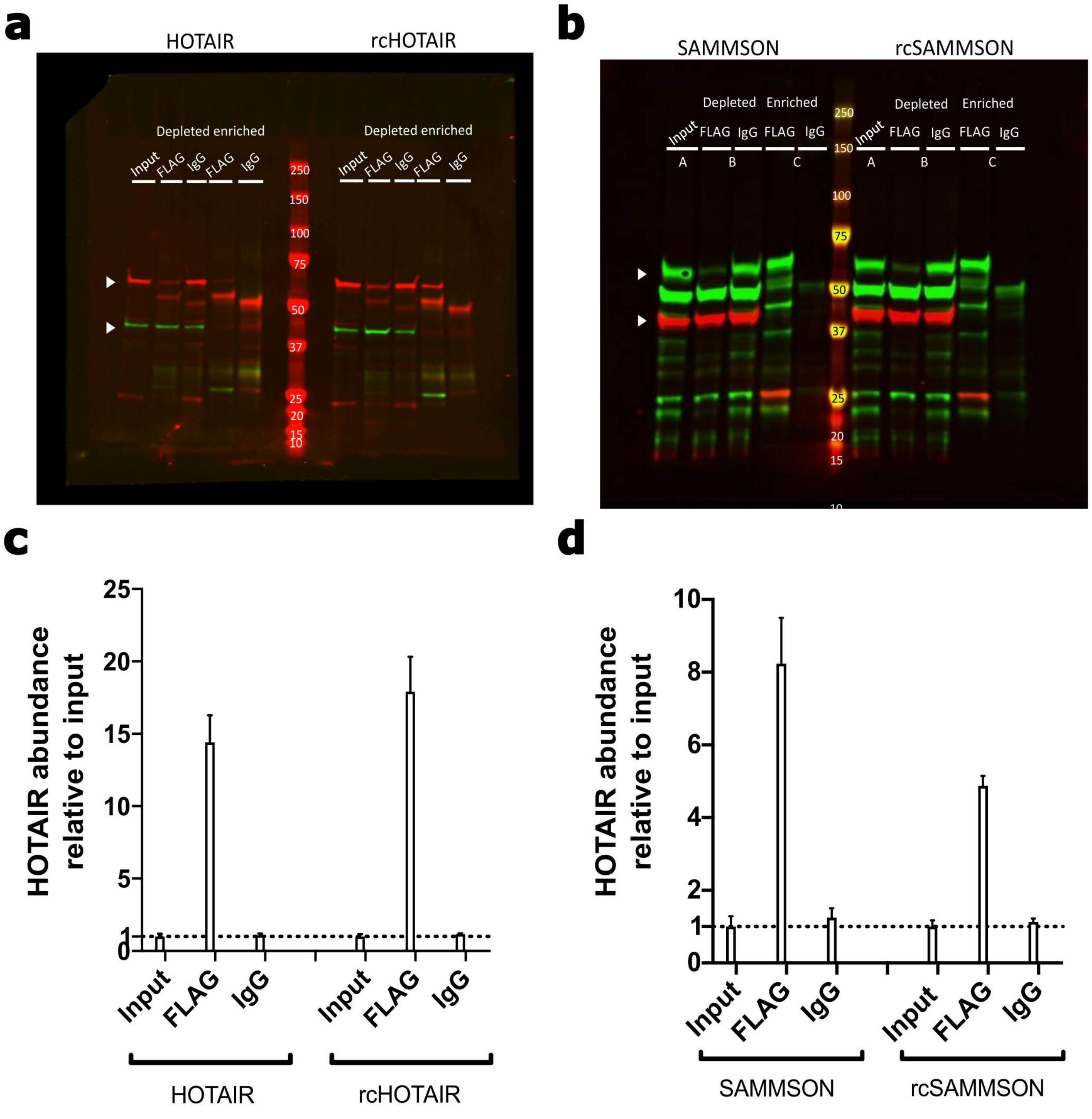

Figure S3: Validation of MCP-BirA* recruitment to 12X MS2-tagged lncRNA by RIP. Western blot analysis showing input (1%), depleted and enriched sample (10%) of **(a)** HOTAIR and rcHOTAIR, and **(b)** SAMMSON and rcSAMMSON after 24 h doxycycline induction. White arrows indicate MCP-BirA*-FLAG (62.5 kDa) and ACTB (42 kDa; internal control). RNA enrichment in the enriched fractions of the same samples was detected by RT-qPCR in the same samples for **(c)** HOTAIR and rcHOTAIR, and **(d)** SAMMSON and rcSAMMSON. RNA enrichment was calculated by normalization to housekeeping genes UBC, YWHAZ, and SDHA. Signal was subsequently scaled relative to the input (1%) fraction. IgG isotype control samples were used to assess aspecific binding.

Supplementary Figure 4

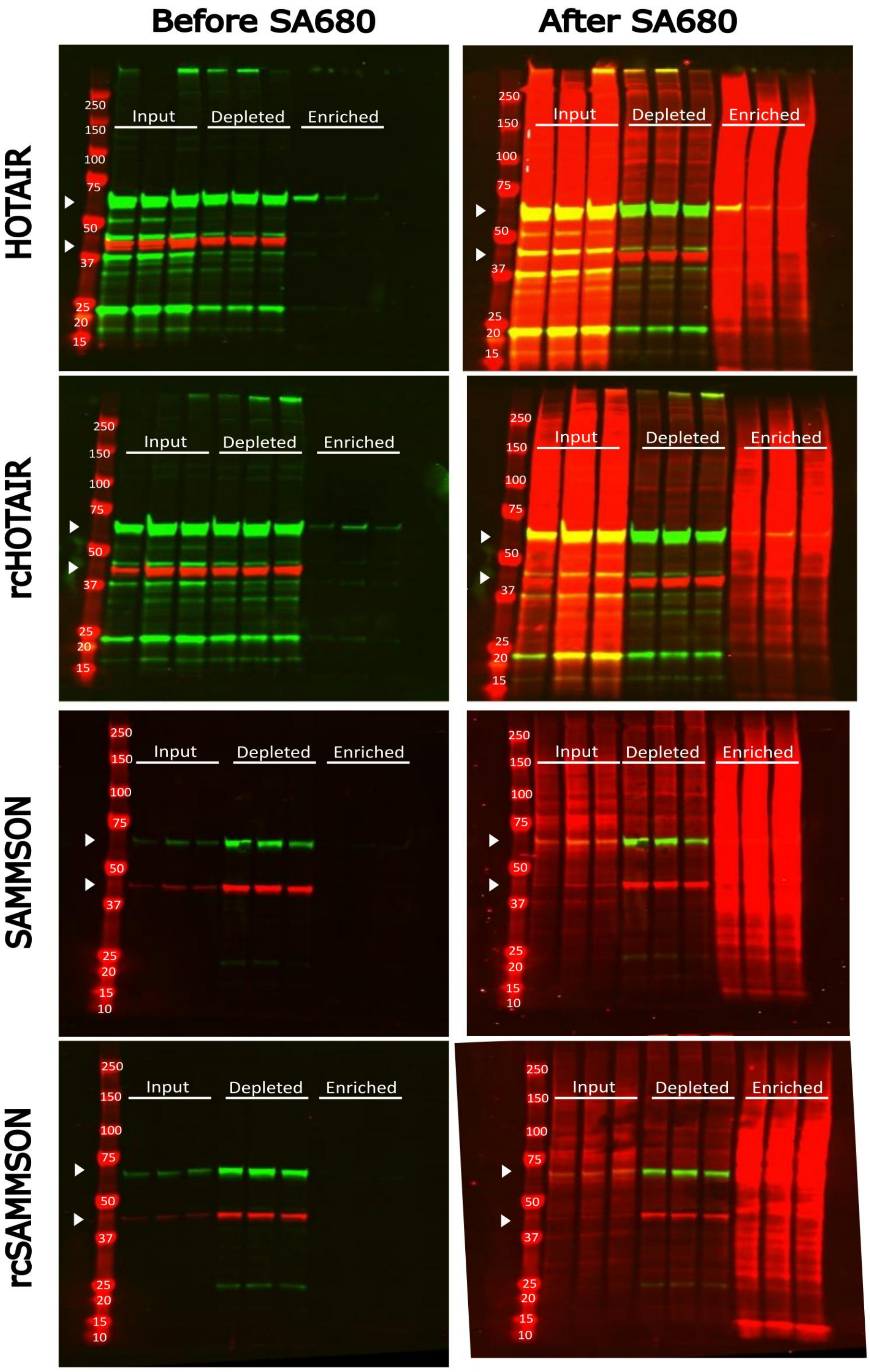

Figure S4: Assessment of biotinylation in the RNA-BioID samples prior to LC-MS/MS analysis. Uncropped western blots of input, depleted, and enriched fractions for all triplicate MS samples for HOTAIR, rcHOTAIR, SAMMSON, and rcSAMMSON. Blots were stained first for MCP-BirA*-FLAG (62.5 kDa) to assess doxycycline-induced expression and ACTB (42 kDa) as a lysis control marker, and subsequently stained with streptavidin (SA680) to assess biotinylation.

Supplementary Figure 5

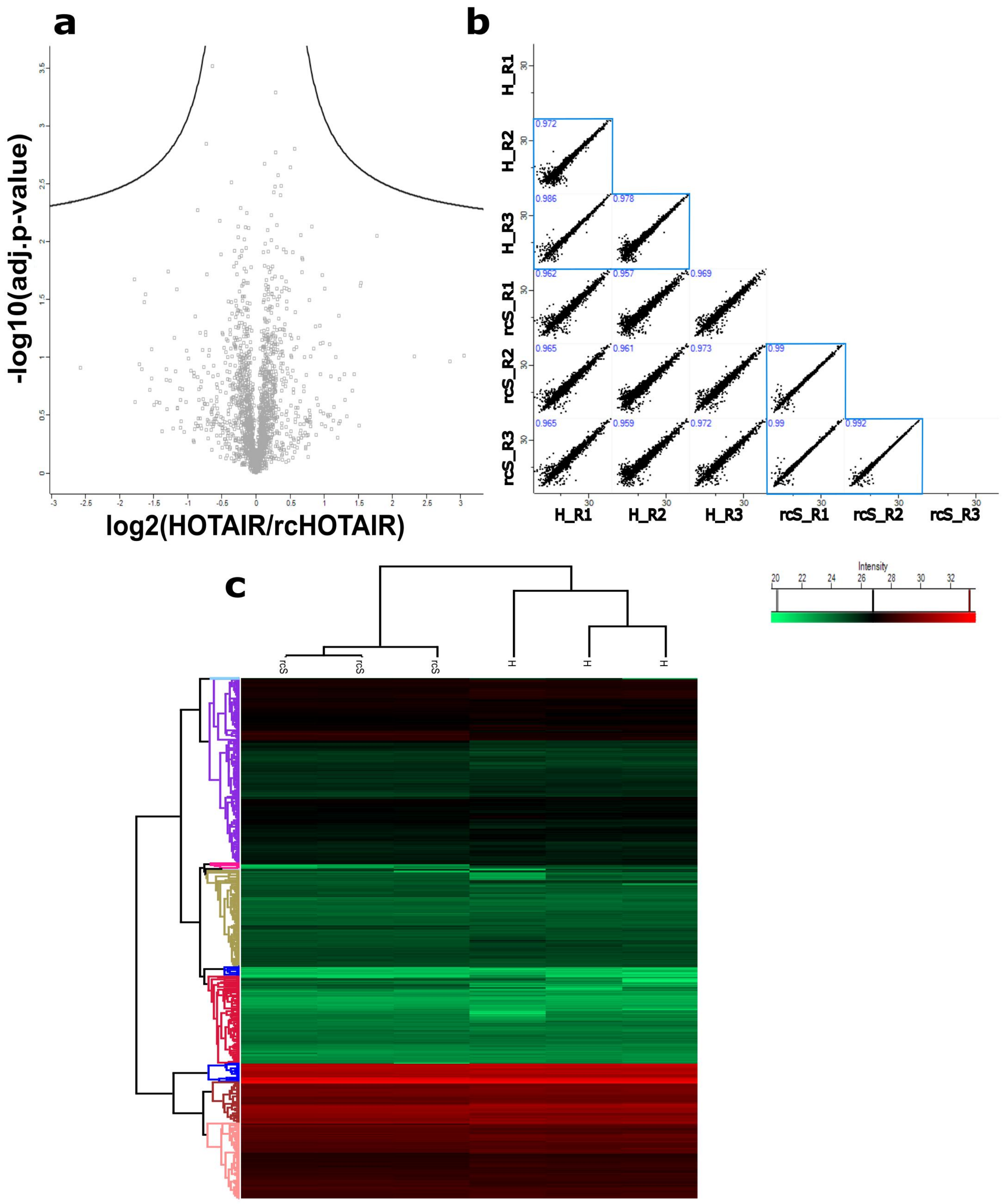

Figure S5: Quality controls for RNA-BioID samples. **(a)** Volcano plot showing the HOTAIR/rcHOTAIR analysis (FDR 0.05; s0 0.1). **(b)** Scatterplot showing the Pearson correlation coefficient of all replicates. **(c)** Heatmap showing all proteins identified in the HOTAIR and rcSAMMSON samples. LFQ intensities of rows and columns were preprocessed with k-means and clustered based on the average-linkage.

Supplementary Figure 6

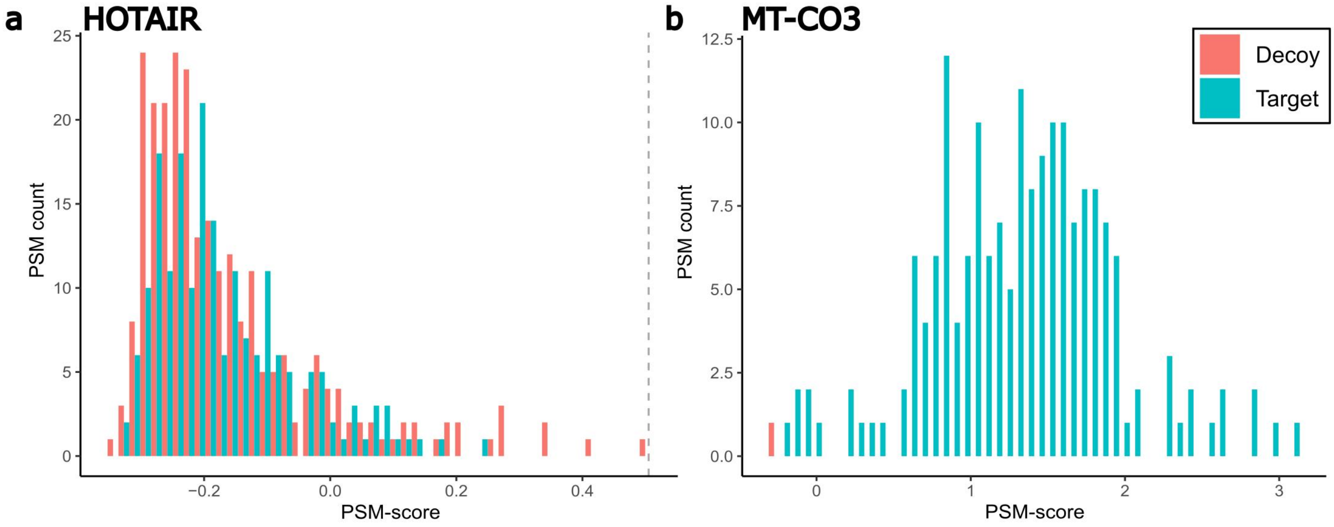

Figure S6: Distribution of peptide-spectrum matches (PSMs) found in PRIDE proteomics datasets of *in silico* predicted peptides for HOTAIR **(a)**, MT-CO3 **(b)**, and decoy databases. PSM-score was determined by Ionbot. Dashed line represents FDR 0.01.

Supplementary Figure 7

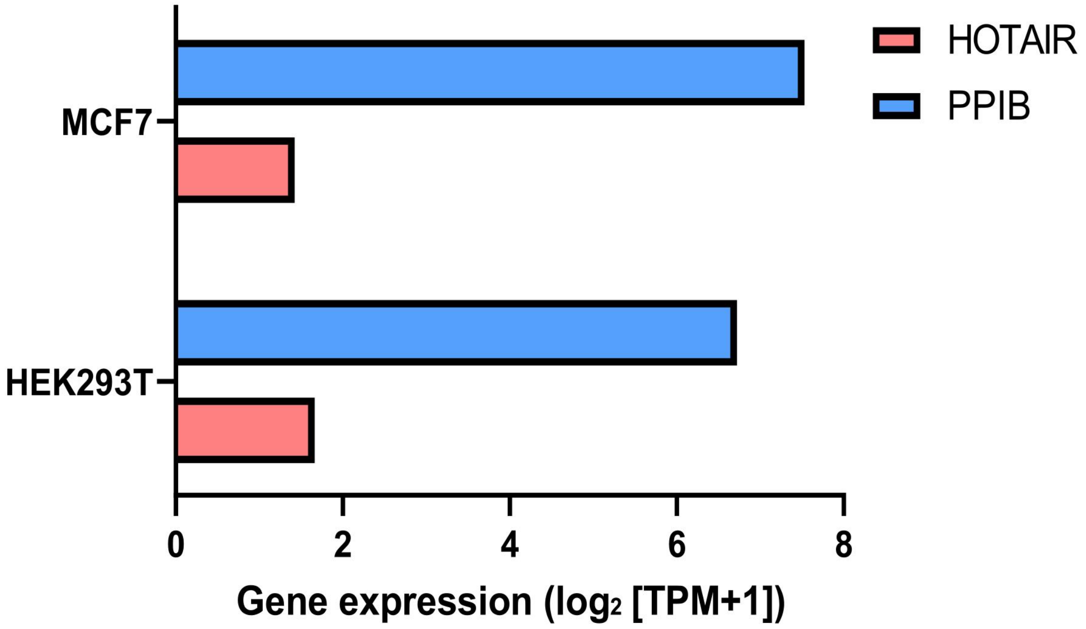

Figure S7: RNA Atlas expression profiles. RNA-sequencing (TPM) of HOTAIR and PPIB in HEK293T and MCF7 cells.
